# Claudin-4 activity in ovarian tumor cell apoptosis resistance and migration

**DOI:** 10.1186/s12885-016-2799-7

**Published:** 2016-10-11

**Authors:** Douglas A. Hicks, Carly E. Galimanis, Patricia G. Webb, Monique A. Spillman, Kian Behbakht, Margaret C. Neville, Heidi K. Baumgartner

**Affiliations:** 1 Division of Reproductive Sciences, Department of Obstetrics and Gynecology, University of Colorado Denver, Anschutz Medical Campus, Mail Stop 8613, 12700 E. 19th Avenue, Aurora, Colorado 80045 USA; 2 Texas Oncology, Sammons Cancer Center, Baylor University Medical Center, 3410 Worth Street, Dallas, Texas 75246 USA

**Keywords:** Claudin-4, Ovarian cancer, Apoptosis, Motility

## Abstract

**Background:**

Claudin-4 is a transmembrane protein expressed at high levels in the majority of epithelial ovarian tumors, irrespective of subtype, and has been associated with tumor cells that are both chemoresistant and highly mobile. The objective of this study was to determine the functional role that claudin-4 plays in apoptosis resistance and migration as well as the therapeutic utility of targeting claudin-4 activity with a small mimic peptide.

**Methods:**

We examined claudin-4 activity in human ovarian tumor cell lines (SKOV3, OVCAR3, PEO4) using in vitro caspase and scratch assays as well as an in vivo mouse model of ovarian cancer. Claudin-4 activity was disrupted by treating cells with a small peptide that mimics the DFYNP sequence in the second extracellular loop of claudin-4. Claudin-4 expression was also altered using shRNA-mediated gene silencing.

**Results:**

Both the disruption of claudin-4 activity and the loss of claudin-4 expression significantly increased tumor cell caspase-3 activation (4 to 10-fold, respectively) in response to the apoptotic inducer staurosporine and reduced tumor cell migration by 50 %. The mimic peptide had no effect on cells that lacked claudin-4 expression. Female athymic nude mice bearing ZsGreen-PEO4 ovarian tumors showed a significant decrease in ovarian tumor burden, due to increased apoptosis, after treatment with intraperitoneal injections of 4 mg/kg mimic peptide every 48 h for three weeks, compared to control peptide treated mice.

**Conclusion:**

Claudin-4 functionally contributes to both ovarian tumor cell apoptosis resistance and migration and targeting extracellular loop interactions of claudin-4 may have therapeutic implications for reducing ovarian tumor burden.

## Background

Claudin-4 is a transmembrane protein that interacts with other claudin-4 proteins expressed at the surface of neighboring cells through extracellular loop interactions [1, 2]. These interactions of claudin-4 have been most well defined in normal epithelia in culture, where claudin-4 is often restricted to tight junction structures and has been shown to provide a barrier to paracellular diffusion [3, 4]. For these reasons, claudin-4 is widely accepted as a differentiation marker and its expression is thought to signify a more epithelial phenotype. The loss of tight junction structure and function is thought to contribute to tumor development [5]. It is therefore somewhat paradoxical that claudin-4 is highly expressed in many types of epithelial tumors including ovarian, breast, gastric, hepatic, pancreatic and other cancers of epithelial origin [6–14]. In fact, claudin-4 is expressed at higher levels in most tumor cells compared to the normal epithelium of origin [15]. Additionally, claudin-4 is often found outside of tight junction structures, often along basolateral membranes, in normal epithelium. These observations suggest that claudin-4 may have functions outside of its traditional barrier forming role in tight junctions and may even participate in activities attributed to the more mesenchymal-like behavior of tumor cells.

In epithelial ovarian cancer, it has been demonstrated that claudin-4 is highly expressed by tumors of all subtypes, grades, and stages [16]. Additionally, claudin-4 is highly expressed in ovarian tumor cell lines categorized in each of the five different molecular subtypes of epithelial ovarian cancer recently described by Tan and colleagues, which includes epithelial- [OVCA429, CaOV3], mesenchymal- [PEO1, SKOV3], and stem-like [OVCAR3, OVCAR5] subtypes [16–18]. These observations suggest that claudin-4 expression may be enhanced early in epithelial ovarian tumor development and its effects are shared among most ovarian tumor cells. It is possible that the activity of claudin-4 in tumor cells may, in fact, stem from their function in the normal epithelium from which the tumor arises and this activity is enhanced due to loss of regulation.

Significant associations between high claudin-4 expression and more aggressive tumor cell behavior have been reported by several laboratories. Stewart and colleagues found that claudin-4 was one of the most highly upregulated (7.5-fold increase) proteins expressed by cisplatin resistant IGROV-1 ovarian tumor cells compared to their cisplatin-sensitive counterparts [19]. Recent work in the Mor and Santin laboratories has shown that high claudin-4 gene and protein expression are found in a population of CD44 positive (CD44^+^) ovarian tumor cells exhibiting stem cell-like properties as well as resistance to carboplatin and paclitaxel [20, 21]. Of importance, the CD44^+^ population has significantly higher levels of claudin-4 compared to CD44 negative (CD44^−^) ovarian tumor cells isolated from the same tumor [21]. When either population was injected into the peritoneal cavity of mice, only the CD44^+^ (high claudin-4 expressing tumor cells) exhibited a strong migratory potential [22]. Complementing this work, Janzen and colleagues have recently demonstrated a stem-like population that lacks the expression of the ovarian cancer antigen CA125 [23]. This CA125-negative population is resistant to platinum treatment and can give rise to recurrent disease. The expression of claudin-4 mRNA in this stem-like population of tumor cells is significantly higher than the CA125-positive tumor cell population that is sensitive to platinum treatment. Together, these observations provide evidence that high claudin-4 expressing ovarian tumor cells have the tumor regenerating properties of cancer stem cells and are associated with cisplatin resistance and enhanced migration. These data raise the question of the functional role of claudin-4 in ovarian tumors. Does claudin-4 increase the motility and survivability of ovarian and perhaps other types of tumor cells?

To begin to answer this question we investigated the functional significance of claudin-4 in ovarian tumor cells both in vitro and in vivo. We have previously described a peptide containing five amino acids that mimic a sequence (DFYNP) in the second extracellular loop of claudin-4 that was designed to interrupt claudin-claudin interactions between neighboring epithelial cells. Treatment of normal mammary epithelial cells in culture with the DFYNP peptide disrupted localization of claudin-4 and lead to the induction of apoptosis [24]. Treatment with this peptide could also disrupt migration of these epithelial cells when type I collagen was present in the extracellular matrix (but not when non-physiological cell adhesive or fibronectin was present), suggesting the claudin-4 may also interact with specific components of the extracellular environment, such as matrix proteins, through the second extracellular loop to alter cell behavior. To test our hypothesis that the novel activities we have described for claudin-4 in normal epithelial cells are functionally contributing to ovarian tumor cell behavior, we used the DFYNP peptide as well as silencing of claudin-4 gene expression to examine changes in apoptotic response and migration of ovarian cancer cells. We have found, in tumor cells in culture, that treating with DFYNP or silencing claudin-4 expression can increase tumor cell sensitivity to apoptosis and decrease motility. In a mouse xenograft model of ovarian cancer we found that disruption of claudin-4 with DFYNP can significantly decreases tumor burden. Our results suggest that claudin-4 functionally contributes to ovarian tumor cell apoptosis resistance and migration and that disrupting these functions can reduce ovarian tumor burden in vivo.

## Methods

### Cell culture

SKOV3, OVCAR3, and Zs-Green PEO4 human ovarian tumor cells lines were cultured in RPMI-1640 medium (Gibco, ThermoFisher Scientific, Grand Island, NY, USA) plus 10 % heat-inactivated Fetal Bovine Serum (Access Cell Culture, Vista, CA, USA) and 1 % penicillin/streptomycin (Gibco, ThermoFisher Scientific). Zs-Green PEO4 cells [25] were grown in the presence of 200 μg/ml G418 (Research Products International, Mt Prospect, IL, USA) to maintain only cells expressing Zs-Green. Cells were trypsinized (0.25 % Trypsin, EDTA, Mediatech) and plated 1:3 every 3–4 days. All cell lines were generously provided by Monique A. Spillman and last authenticated in November 2015 by short tandem repeat profiling, as previously described [26]. For treatment and imaging, cells were plated at 1 × 10^4^ cells/well onto Lab-Tek glass 8-chamber slides (NUNC, Rochester, NY) and treated upon confluence (approximately 3 day post plating).

### shRNA knockdown

SKOV3 cells were plated at 3.2 × 10^4^ in a 96-well plate and incubated at 37 °C for 24 h. When cells were 70 % confluent, 1–15 μl of claudin-4 shRNA (TRC#: TRCN0000116627, TRCN0000116628, or TRCN0000116629) or control shRNA (SHC001, pLK0.1-puro Empty Vector) lentiviral suspension (Sigma-Aldrich MISSION® shRNA, University of Colorado Functional Genomics Facility, Boulder, CO, USA) was added to each well and cells were incubated overnight at 37 °C. Medium was then removed and replaced with fresh medium without lentivirus. After 24 h, the cells were treated with 0.5 μg/ml puromycin to select for transduced cells. Colonies of cells were selected and expanded for experiments. Western blot analysis was performed to select cells with the most significant reduction in claudin-4 expression (TRCN0000116627, 10 μl suspension added to cells).

### Caspase-3 activation assay

Apoptosis was measured by caspase-3 activation. After treatment of tumor cells with 2 mM staurosporine (Sigma-Aldrich, St Louis, MO, USA), 400 μM claudin mimic peptide (NH_2_-GDFYNPG-OH, D-amino acid conformation, University of Colorado Protein and Peptide Chemistry Core), or 400 μM inactive control peptide (NH_2_-GDGYNPG-OH, D-amino acid conformation, University of Colorado), cells were fixed with 10 % phosphate buffered formalin (Fisher Scientific, Pittsburgh, PA, USA) at room temperature (RT) for 15 min. Cells were washed with phosphate buffered saline (PBS) before being permeabilized with 0.5 % Triton X-100 for 5 min, washed with PBS, blocked with 2 % bovine serum albumin (BSA) for 1 h, and treated with primary antibody directed to cleaved caspase-3 (1:400; rabbit anti-cleaved caspase-3, Cell Signaling, Danvers, MA, USA) overnight at 4 °C. Cells were washed with PBS five times before application of secondary antibody conjugated to a fluorescent probe (1:100; donkey anti-rabbit CY5, Jackson ImmunoResearch Laboratories, West Grove, PA, USA) and 5 μg/ml 4′,6-diamidina-2-phenylindole (DAPI; Sigma-Aldrich) for 45 min at RT followed by five washed with PBS. OPDA (20 mg/ml, o-phenylenediamine dihydrochloride in 1 M Tris, pH 8.5) was applied to slides for preservation of fluorescence and coverslip mounted. Fluorescence was imaged with an Olympus Spinning Disk confocal microscope (University of Colorado AMC Light Microscopy Core) and images analyzed for percent of cell population positive for active caspase-3, using SlideBook software (Intelligent Imaging Innovations, Inc., Denver, CO, USA).

### Motility assay

A scratch assay was used to measure changes in cell migration. Monolayers of SKOV3 or OVCAR3 tumor cells were cultured on glass chamber slides (as described above) coated with type I collagen (100 μg/ml; Sigma-Aldrich). Once cell layers reached confluence, the monolayer was scratched with a 200 μl pipette tip, creating a vertical cell-free gap. Media was immediately changed to normal growth media or growth media plus 400 μM NH_2_-G*DFYNP*G-OH or an inactive control peptide NH_2_-G*DF* *G* *NP*G-OH [27]. One set of slides was immediately fixed. Another set of slides was incubated at 37 °C for 8 h (SKOV3) or 18 h (OVCAR3) before fixing with 10 % phosphate buffered formalin for 15 min at RT. Cells were treated with Alexa Fluor® 647 Phalloidin (Life Technologies, Carlsbad, CA, USA) and DAPI, washed with PBS, coverslipped, and imaged with the Olympus Spinning Disk confocal microscope. Fluorescent images of two fields of interest per well, near the center of each well, were taken and SlideBook software was used to measure the distance across the gap (20 measurements along the length of each imaged scratch were taken for each well under each condition).

### Mouse model of ovarian tumor burden

Changes in tumor burden were monitored using a mouse xenograft model of ovarian cancer [25]. Six to eight-week old ovariectomized athymic NCr-nu/nu female mice (NCI, Charles River Laboratories, USA) were given an intraperitoneal (i.p.) injection of 1X10^7^ Zs-Green PEO4 tumor cells (under isoflurane anesthesia) in addition to a subcutaneous pellet of β-estradiol (2 mg; Sigma-Aldrich). Once tumors had established, approximately 5–7 weeks post injection of tumor cells, mice were treated with i.p. injections of control peptide (8 mg/kg, DGYNP), or DFYNP peptide (4 mg/kg) every 48 h for three weeks. Mice were imaged with a Xenogen IVIS200 imaging system before treatment and after each week of treatment. All animal procedures were performed under the guidelines and approval of the University of Colorado Institutional Animal Care and Use Committee. Changes in photon flux in a defined region of the mouse abdomen (2.80 cm × 2.80 cm) were measured using Living Image 2.60 software. After treatment, mice were sacrificed, the abdomen of each mouse was opened and the peritoneal cavity was imaged with a Canon® EOS Rebel T3I digital camera. Remaining tumors were collected and either snap-frozen in liquid nitrogen and stored at −80 °C or fixed in 10 % phosphate buffered formalin overnight in 4 °C and taken to the University of Colorado Histology Core for paraffin embedding. Sections of tissue were placed on glass slides for immunofluorescence analysis.

### Immunofluorescence for mouse tissue sections

Paraffin embedded sections of tumor tissue were immersed in xylene for 20 min, twice, to remove paraffin and then hydrated in decreasing amounts of ethanol (2 washes in 100 % ethanol, one wash in 70 % ethanol, and one wash in 30 % ethanol) for three minutes each. Sections were then washed in PBS two times for 5 min each before antigen retrieval, by boiling sections in citrate-based Antigen Unmasking Solution (Vector Laboratories, Inc., Burlingame, CA, USA) for 10 s followed by a 45 s rest and repeated 10 times. Sections were cooled for 10 min before washing with PBS and permeabilizing with 1 % Triton X-100 for 15 min. After two washes with PBS, sections were blocked for one hour with 10 % normal donkey serum plus 10 μM/ml saponin. Sections were then treated with antibodies to detect cleaved caspase-3 as described above.

### Immunohistochemistry for mouse tissue sections

Paraffin embedded sections of tumor tissue were baked for 30 min at 60 °C and then immersed in xylene twice, for 5 min, to remove paraffin and then hydrated in decreasing amounts of ethanol (twice in 100 %, once in 95 %, and once in 70 %) for one minute each and washed in deionized water (dH_2_O) for 1 min. Antigen retrieval was performed by immersing slides in 10 mM citrate buffer (in dH_2_O, pH 6.0) and heating at 120 °C (20–25 psi) for 5 min in a Biocare Medical Decloaker. Slides were then washed three times in dH_2_O before immersion in 3 % hydrogen peroxide. Slides were then washed three times in dH_2_O and then placed in Tris Buffered Saline with 0.025 % Tween (TBST) for two minutes. Slides were blocked in 10 % normal goat serum (in TBST) for 30 min at room temperature before applying the claudin-4 (1:200) or caspase-3 (1:200) antibodies, described above, and incubating overnight at 4 °C. Slides were washed three times with TBST for two minutes each wash. DAKO Envision anti-rabbit secondary (DAKO, Carpentina, CA, USA) was then applied for 30 min at room temperature before rinsing three times in TBST for 2 min each rinse. Slides were treated with 3,3′ Diaminobenzidine (DAB) for 10 min, rinsed three times with dH_2_O, and counterstained with hematoxylin for 2 min. Slides were washed with dH_2_O for five minutes before application of Permount (Fisher Scientific), coverslipping and imaging.

### Statistics

Data are presented as mean ± standard error of the mean (s.e.m.). An unpaired Student′s *t* test was used for statistical comparison between control and treatment groups. A *p* value of < 0.05 was considered significant.

## Results

### Effect of interfering with Claudin-4 activity and expression on ovarian tumor cell sensitivity to apoptosis

**Fig. 1:**
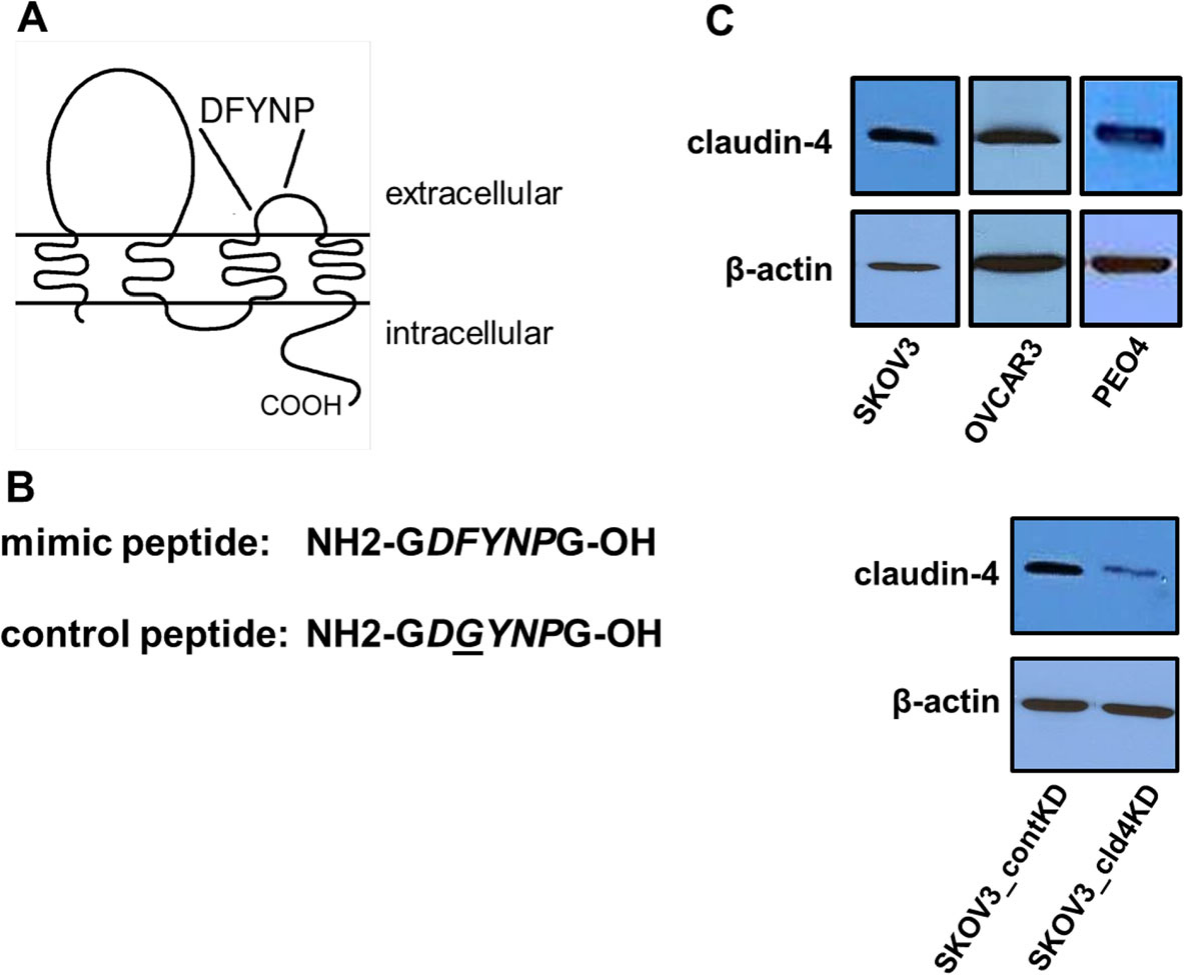
Disruption of claudin-4. Claudin-4 structure (**a**). Claudin-4 is a tetraspanin molecule with two extracellular loops. The second extracellular loop of claudin-4 contains the DFYNP sequence that is targeted by our small DFYNP mimic peptide (**b**). An inactive peptide with a glycine substitution for phenylalanine was used as a control peptide. Claudin-4 expression was reduced in ovarian tumor cells treated with claudin-4 shRNA. Representative Western blot analysis of claudin-4 expression (**c**), and β-actin as a loading control, was performed on lysates from SKOV3, OVCAR3, and PEO4 cells. SKOV3 cells were treated with control empty vector shRNA lentiviral suspension or claudin-4 shRNA lentivirus for 18 h before washing cells and treating with puromycin. Control shRNA treatment did not alter claudin-4 expression and claudin-4 shRNA drastically reduced claudin-4 expression compared to SKOV3 cells not treated with shRNA

To investigate the functional contribution of claudin-4 to ovarian tumor cell behavior, we disrupted claudin-4 in two ways. First, we disrupted claudin-4 with a previously described peptide that mimics a specific sequence in the second extracellular loop of claudin-4, DFYNP (Fig. 1a, b). This peptide is in the D-amino acid conformation, to enhance stability, and has no toxic effects in mice up to the highest dose tested (32 mg/kg, data not shown), a dose 8-fold higher than used in this study. An inactive DGYNP peptide was used as a control (Fig. 1b). Western blot analysis revealed that native SKOV3, OVCAR3, and PEO4 ovarian tumor cells expressed claudin-4. We then disrupted claudin-4 in the SKOV3 cell line by using shRNA-mediated gene silencing. Western blot analysis confirmed that claudin-4 protein expression in claudin-4 shRNA-treated SKOV3 cells, “SKOV3_cld4KD”, were reduced compared to empty vector control shRNA-treated, “SKOV3_contKD” (Fig. 1c).

**Fig. 2:**
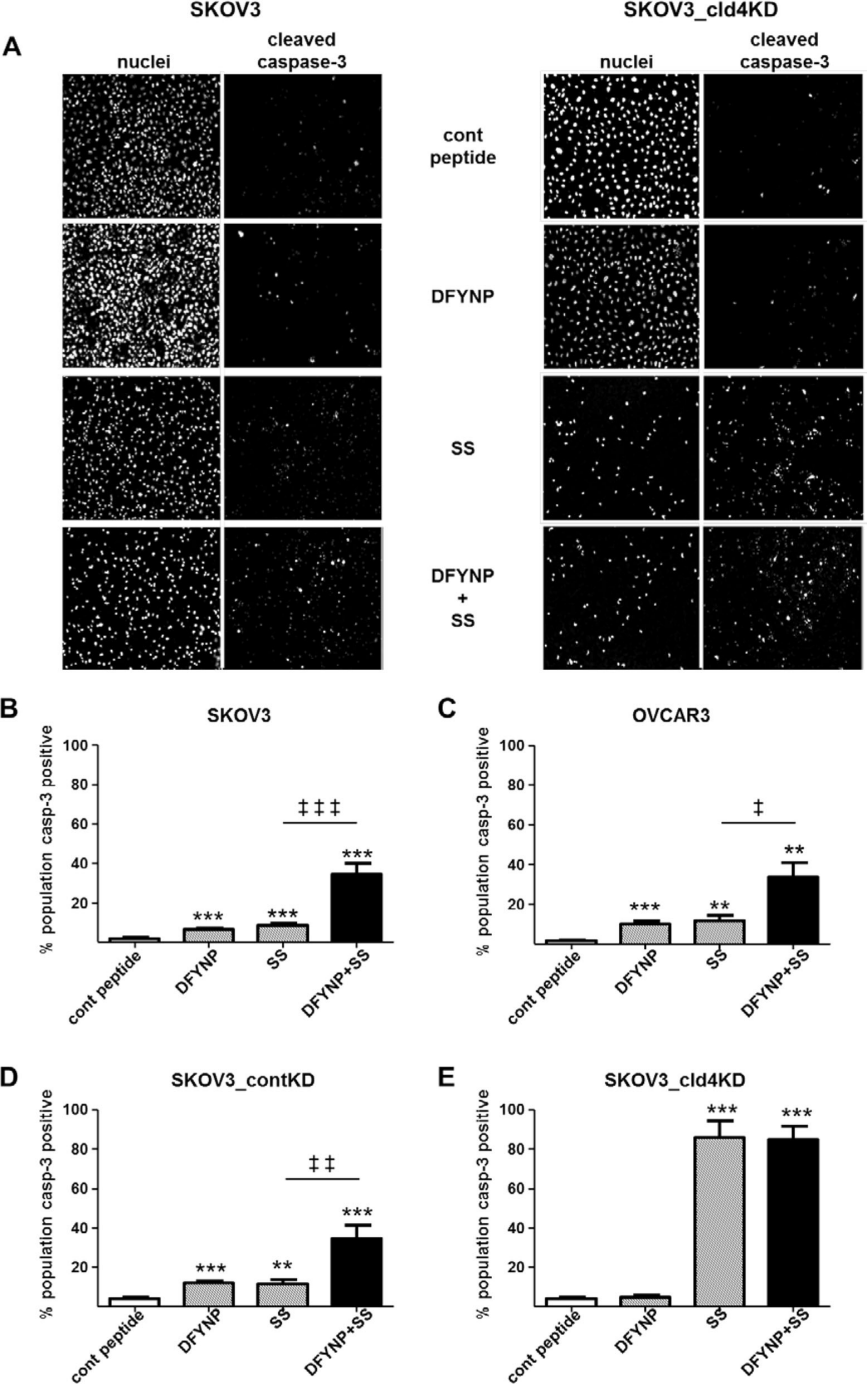
Claudin-4 promotes tumor cell survival. SKOV3, OVCAR3, SKOV3_contKD, and SKOV3_cld4KD cells were treated with 400 μM control peptide (“cont peptide”), 400 μM claudin mimic peptide (“DFYNP”), 2 μM staurosporine (“SS”, known inducer of apoptosis) or DFYNP + staurosporine (“DFYNP + SS”) for 18 h before being fixed and stained with dapi (to identify nuclei) and fluorescent antibody directed to active caspase-3. Representative fluorescent images of nuclei (dapi staining) and activated caspase-3 for each treatment group are shown for the claudin-4 expressing and claudin-4 knockdown SKOV3 cells (**a**). Percent of cell population positive for casp-3 activation was plotted (**b-e**). Mean ± s.e.m, *n* = 4 per treatment group, **p < 0.01, ***p < 0.001 vs. control peptide treated and ^‡^ *p* < 0.02, ^‡‡^ *p* < 0.01, ^‡‡‡^ *p* < 0.001 for staurosporine only vs. DFYNP plus staurosporine treatment

We treated SKOV3 human ovarian tumor cells with 400 μM DFYNP for 18 h, finding that disruption of the second extracellular loop of claudin-4 significantly increased caspase-3 activation (6.7 ± 0.5 % cells caspase-3 positive) compared with cells treated with the inactive control peptide (1.9 ± 0.6 % positive cells, *p* = 0.0002, Fig. 2a, b). Treatment of these cells with 2 μM staurosporine, a known inducer of apoptosis, induced a level of apoptosis (8.7 ± 0.9 %, *p* < 0.0001) at 18 h similar to that observed with DFYNP. Pretreatment of the SKOV3 cells with DFYNP before treatment with staurosporine significantly enhanced the apoptotic response to 34.6 ± 5.5 %, a nearly 4-fold increase in caspase-3 activation compared to cells treated with staurosporine alone (*p* = 0.0004). OVCAR3 cells showed a similar response to DFYNP and/or staurosporine as the SKOV3 cells (Fig. 2c). The level of apoptosis induced by DFYNP plus staurosporine in these cells (33.9 ± 7.3 % caspase-3 positive cells) was, again, significantly higher than that induced by either DFYNP (10.2 ± 1.4 %, *p* < 0.01) or staurosporine alone (11.7 ± 2.7 %, *p* = 0.0174).

If DFYNP is indeed acting through an effect on claudin-4, knocking down this molecule with shRNA should abrogate any effect on apoptosis. Further, we predict that knockdown of claudin-4 would increase the sensitivity of the cells to an apoptosis inducing agent. These hypotheses were tested by using our claudin-4 knockdown cells (SKOV3_cld4KD). Fig. 2d, e show that control knockdown cells behaved in the same manner as our native SKOV3 and OVCAR3 cells, with DFYNP potentiating the ovarian tumor cell apoptotic response to staurosporine (from 11.6 ± 2.2 to 34.6 ± 6.6 %, *p* = 0.0054). The claudin-4-targeting DFYNP peptide did not increase caspase-3 activation in the SKOV3_cld4KD cells (4.8 ± 0.9 %) compared to control peptide treated (3.9 ± 0.8 %, *p* = 0.5217). In addition, the claudin-4 knockdown cells (SKOV3_cld4KD) were more sensitive to staurosporine alone (85.7 ± 8.5 % population caspase-3 positive) compared to the claudin-4 expressing native SKOV3 cells and SKOV3 cells treated with control shRNA (11.6 ± 2.3 %, *p* < 0.0001). DFYNP did not enhance apoptotic response to staurosporine in the SKOV3_cld4KD cells (84.6 ± 6.9 %, *p* = 0.92). These results show that interfering with claudin-4 activity or loss of claudin-4 expression significantly increases sensitivity of tumor cells to cellular apoptosis and that DFYNP is active only when claudin-4 is expressed.

### Claudin-4 facilitates tumor cell motility

**Fig. 3:**
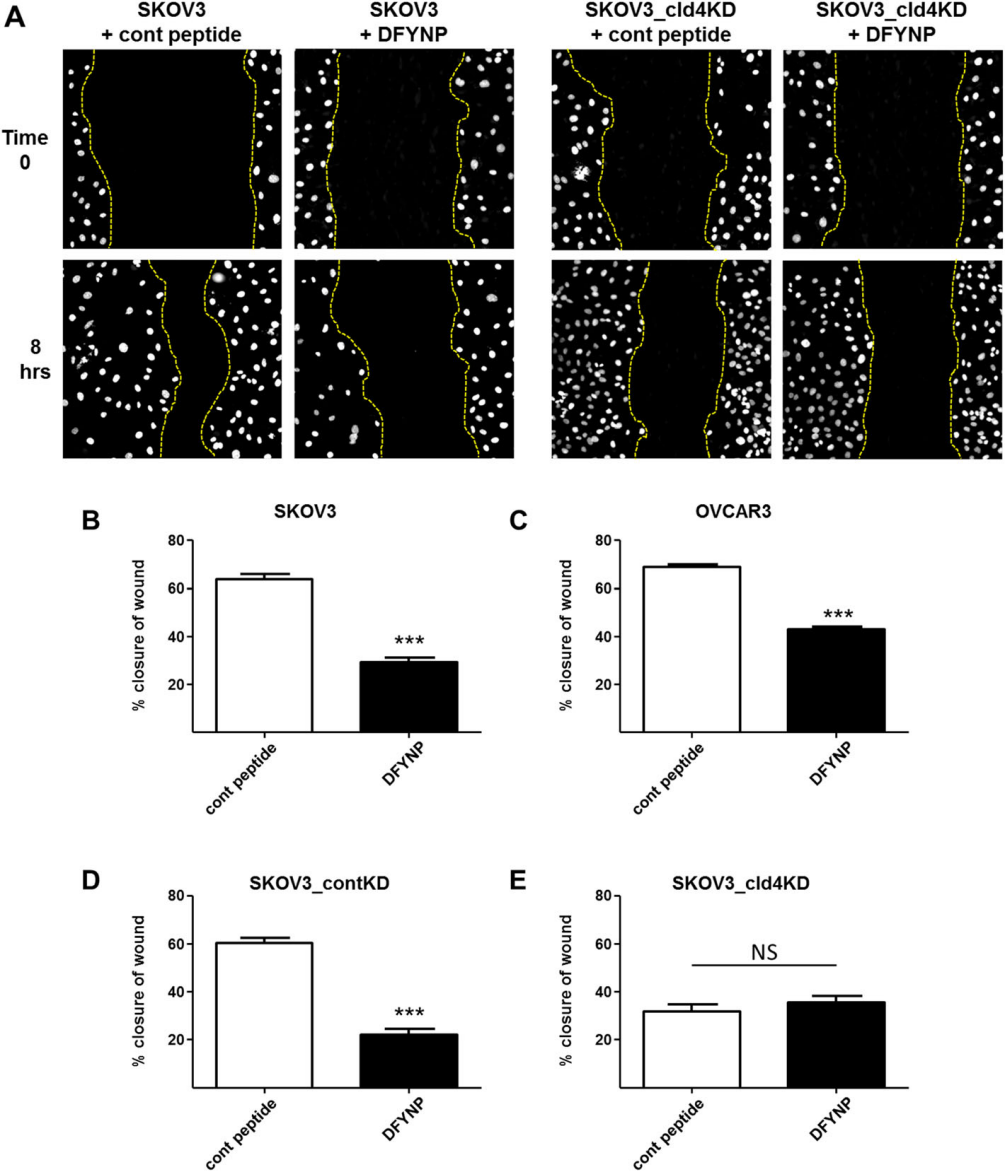
Disrupted claudin-4 diminishes ovarian tumor cell motility. Monolayers of SKOV3 (**a**, **b**) and OVCAR3 (**c**) cells or SKOV3 control (**d**) or claudin-4 knockdown (**a**, **e**), via shRNA, were scratched and then incubated at 37 °C in the presence of 400 μM control peptide (“Cont peptide”) or 400 μM claudin mimic peptide (“DFYNP”) for 8 (SKOV3) or 18 (OVCAR3) hours. Representative fluorescent images of nuclei (dapi staining) of scratched monolayers at time 0 and 8 h post scratch are shown for claudin-4 expressing and claudin-4 knockdown SKOV3 cells (**a**). Distance across the gap remaining was measured immediately following scratching and at 8 or 18 h after treatment using SlideBook software. Yellow dashed lines represent boundaries of wound. Percent closure of the wound was plotted (**b-e**). Mean ± s.e.m, *n* = 4 per treatment group, **p < 0.01, ***p < 0.001 vs. control peptide treated

SKOV3 and OVCAR3 human ovarian tumor cell lines were used to examine the effect of claudin-4 activity and expression on tumor cell motility. Cell monolayers were scratched and cell movement into the wound area was monitored over time. The size of the gap created by the scratch was measured immediately after scratching as well as 8 (SKOV3) or 18 (OVCAR3) hours later. The percentage of wound closure was calculated and plotted (Fig. 3). In the presence of 400 μM control peptide, 63.7 ± 2.2 % of the wound was closed in the SKOV3 monolayers by 8 h. Treatment with 400 μM DFYNP significantly reduced wound closure to only 29.1 ± 2.1 % (*p* < 0.0001) by 8 h (Fig. 3a, b). OVCAR3 cells showed a similar response to DFYNP. Wound closure with control peptide treatment was 68.7 ± 1.1 % compared to 42.8 ± 1.2 % in the presence of DFYNP, (*p* < 0.001, Fig. 3c). Movement of cells into the wound was significantly slower in the SKOV3_cld4KD cells compared to the SKOV3_contKD cells (31.9 ± 2.8 % vs. 60.2 ± 2.3 % closure, *p* < 0.001, Fig. 3d, e). Although the SKOV3_contKD cells showed reduced migration with DFYNP (22.0 ± 2.6 % closure, *p* < 0.0001), SKOV3_cld4KD cells did not respond to DFYNP (35.4 ± 2.9 % closure, *p* = 0.3823 compared to treatment with control peptide). These results provide evidence that claudin-4 functionally contributes to tumor cell motility.

### Disruption of claudin-4 reduces tumor burden

**Fig. 4:**
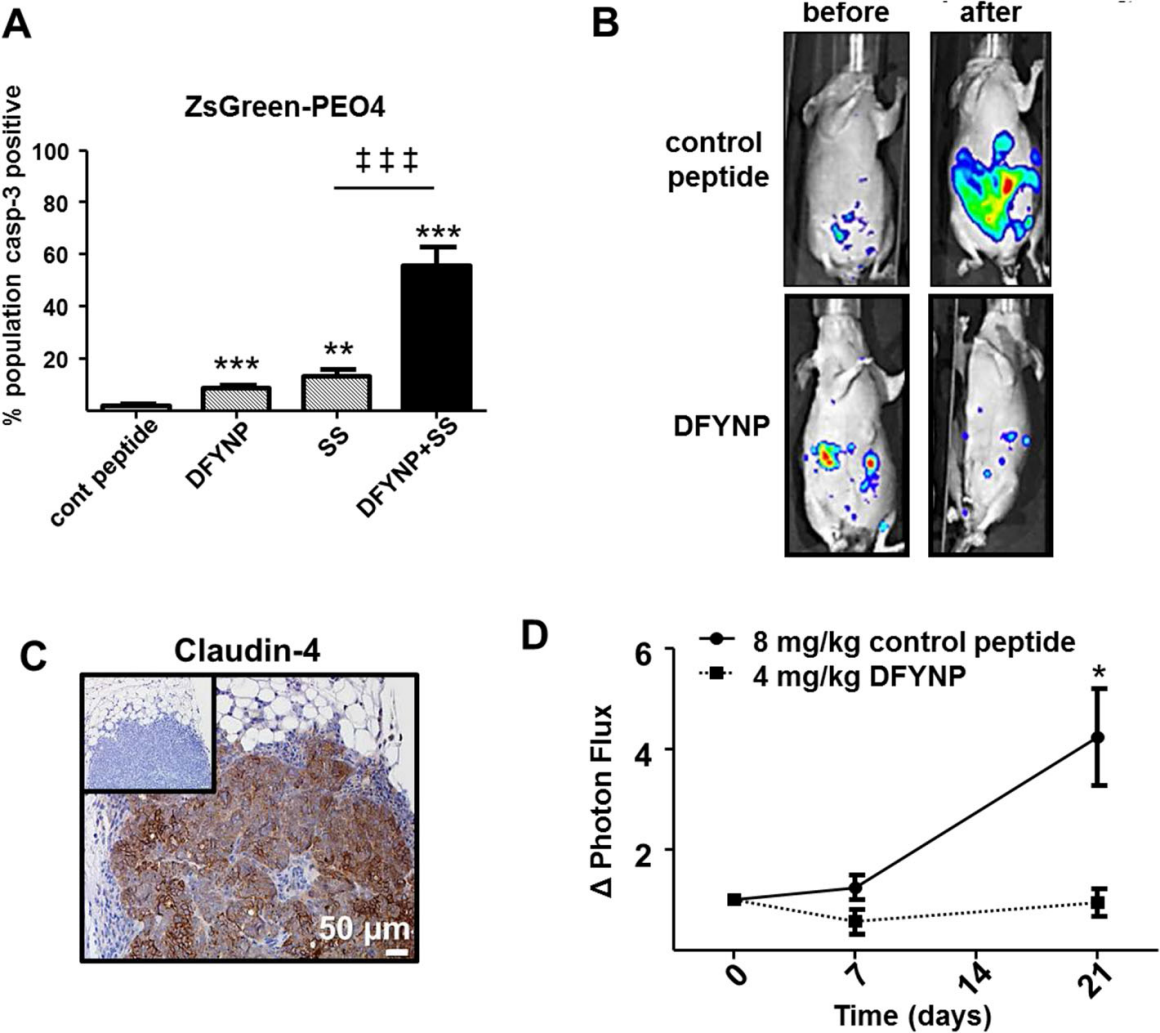
Targeting claudin-4 interactions reduces tumor burden in vivo. ZsGreen-PEO4 ovarian tumor were treated with 400 μM control peptide (“cont peptide”), 400 μM claudin mimic peptide (“DFYNP”), 2 μM staurosporine (“SS”, known inducer of apoptosis) or DFYNP + staurosporine (“DFYNP + SS”) for 18 h before being fixed and stained with dapi (to identify nuclei) and fluorescent antibody directed to active caspase-3. **a** Percent of cell population positive for casp-3 activation was plotted. Mean ± s.e.m, *n* = 4 per treatment group, **p < 0.01, ***p < 0.001 vs. control peptide treated and ^‡‡‡^ *p* < 0.001 for staurosporine only vs. DFYNP plus staurosporine treatment. Female athymic nude mice with fluorescent ZsGreen-PEO4 human ovarian tumors were treated with 4 mg/kg control peptide or 4 mg/kg DFYNP (i.p. injection) every 48 h for three weeks. **b** Representative IVIS images of ovarian tumors taken before and after treatment. **c** Immunohistochemical analysis of claudin-4 expression in ZsGreen-PEO4 tumors in vivo. Inlay shows same section of tissue without primary antibody. **d** Quantitative analysis of change in photon flux, a measure of tumor size, before and after treatment. Mean ± s.e.m, *n* = 8-10 animals per group, *p < 0.05, ***p < 0.001 vs. control-treated

Our next question was whether these findings would translate to tumors in an in vivo model of ovarian cancer. To answer this question we used an established mouse xenograft model of ovarian cancer to examine the contribution of claudin-4 to overall ovarian tumor burden [25]. This model uses fluorescently labeled PEO4 cells (ZsGreen-PEO4) that show an apoptotic response similar to SKOV3 and OVCAR3 cells (Fig. 4a). Female nude mice were given an intraperitoneal (i.p.) injection of ZsGreen-PEO4 cells. After tumors were established, at 7 weeks, mice were given i.p. injections of 4 mg/kg control peptide or 4 mg/kg DFYNP every 48 h for 3 weeks. Tumor burden was imaged with an IVIS 200 Imaging System and photon flux measured for each mouse before treatment and at 7 and 21 days after initiation of treatment (Fig. 4b). Immunohistochemical analysis confirmed that claudin-4 was strongly expressed in the ZsGreen-PEO4 tumors (Fig. 4c). After the first week of treatment, the tumor burden appeared smaller in the DFYNP treated mice, but the difference was not significant (*p* = 0.0729). By three weeks of treatment, however, mice treated with DFYNP had significantly less tumor (1.42 ± 0.24 fold increase in photon flux compared to the start of treatment) than mice treated with control peptide (3.56 ± 0.92 fold increase, *p* = 0.0216, Fig. 4d).

**Fig. 5:**
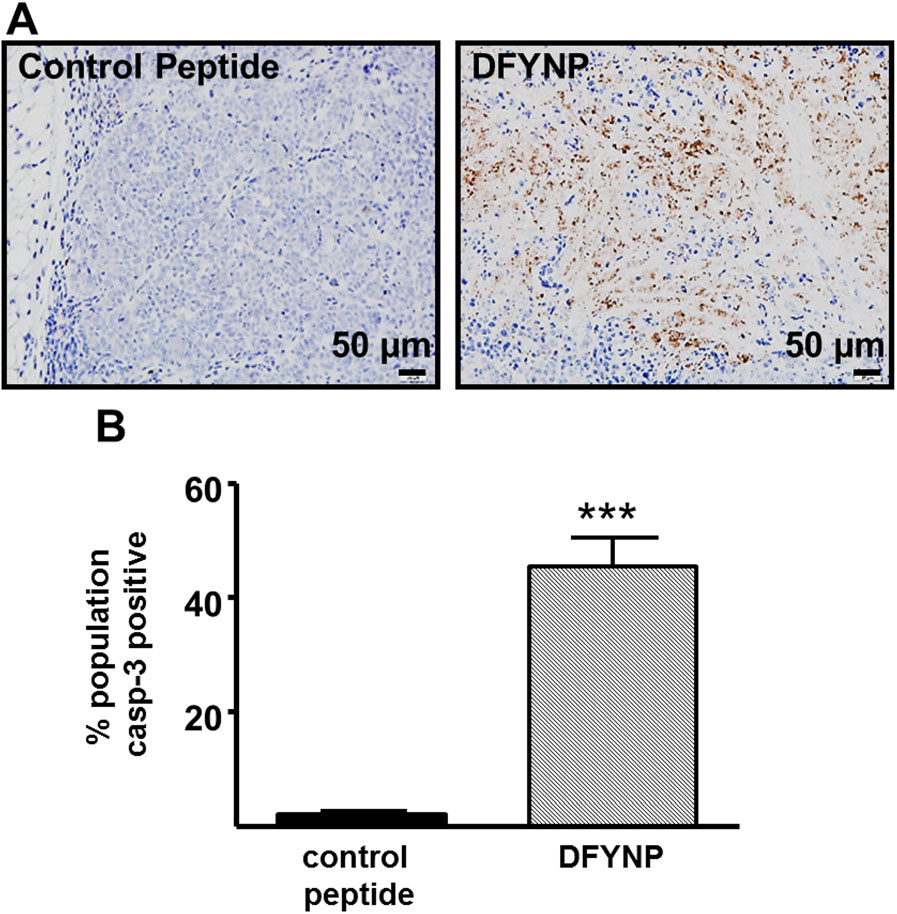
Targeting claudin-4 interactions induces apoptosis in tumors in vivo. Female athymic nude mice with fluorescent ZsGreen-PEO4 human ovarian tumors were treated with 4 mg/kg control peptide or 4 mg/kg DFYNP (i.p. injection) every 48 h for three weeks. Remaining tumors were removed after three weeks of treatment. (**a**) Representative images of cleaved caspase-3 in tumors from mice treated with control peptide vs. DFYNP peptide. (**b**) Percent cell population positive for cleaved caspase-3 was calculated for control peptide and DFYNP treated tumors remaining after treatment. Mean ± s.e.m, *n* = 11-15 animals per group, *p < 0.05, ***p < 0.001 vs. control-treated

Although a decrease in tumor spread with DFYNP treatment can be seen qualitatively (Fig. 4b), motility could not be directly measured in this system. Apoptotic response, however, could be measured by analyzing tumor cells present at the end of the study. Therefore, tumors remaining in treated mice at the end of the study were collected, fixed, and examined by immunohistochemistry and immunofluorescence for caspase-3 activation (Fig. 5). Tumors from mice treated with DFYNP showed significantly more caspase-3 activation (45.35 ± 5.09 % cell population caspase-3 positive) compared to control peptide treated tumors (2.06 ± 0.50 %, *p* < 0.0001).

## Discussion

We have shown that overexpression of claudin-4 reduces tumor cell sensitivity to apoptosis and enhances tumor cell migration. Disruption of extracellular loop activity of claudin-4 in the presence of DFYNP enhanced tumor cell sensitivity to apoptosis, inhibited tumor cell motility, and ultimately led to reduced tumor burden in vivo. The loss of claudin-4 expression in ovarian tumor cells produced the same pro-apoptotic and anti-migratory activity as DFYNP. In cells that lack claudin-4 expression, the peptide did not activate caspase-3 or reduce wound closure. The lack of tumor cell response to DFYNP in claudin-4 knockdown cells indicates that DFYNP is specifically targeting claudin-4. Together these observations suggest that claudin-4 plays a critical role in promoting tumor cell survival and tumor spread, two major contributing factors to poor survival outcomes of ovarian cancer patients.

Others have observed high claudin-4 expression in ovarian tumor cells that are resistant to chemotherapeutic agents such as cisplatin and paclitaxel [19, 21]. We have shown that claudin-4 can decrease tumor cell sensitivity to apoptosis. It is possible, therefore, that claudin-4 may contribute to chemoresistance by inhibiting apoptotic signaling responses. How claudin-4 is inhibiting apoptosis in ovarian tumors is not known, however, we have previously shown interfering with claudin-4 in normal mammary epithelial cells with DFYNP disrupts claudin-4 localization and induces death by interacting with occludin and components of the death inducing signaling complex (DISC) to activate the extrinsic apoptotic pathway [3]. It is possible, therefore, that the transmembrane claudin-4 protein may interact with key molecules near the tumor cell surface to prevent activation of the extrinsic apoptotic signaling pathway. Although the identity of these interacting molecules is still to be determined, it is evident that disruption or loss of claudin-4 can rescue apoptotic response. This finding presents the exciting possibility, and important next-step to test, that disrupting claudin-4 will rescue apoptotic response in ovarian tumor cells that are resistant to cisplatin and paclitaxel. Casagrande and colleagues have demonstrated the *clostridium perfringens* enterotoxin (CPE), recognizing a sequence in the second extracellular loop adjacent to the sequence recognized by DFYNP, is cytotoxic to the high claudin-4 expressing stem-like CD44+ ovarian tumor cells [21]. Although CPE is predicted to act through a different mechanism than DFYNP, effects of both strategies demonstrate the utility of targeting claudin-4 to reduce tumor cell survival. Given the observations that high claudin-4 expression can be found in CD44+ and CA125- stem-like populations and that we can induce apoptosis in tumor cells by disrupting claudin-4, we speculate that targeting claudin-4 activity may be a strategy for eliminating the tumor regenerating population of ovarian tumor cells to prevent recurrent disease.

Agarwal and colleagues have shown that forced expression of claudin-4 in normal human ovarian surface epithelial (HOSE) cells, cells that normally express very little to no claudin-4, increases their ability to migrate [28]. We have shown here that loss of claudin-4 expression in ovarian tumor cells, expressing high levels of claudin-4, can reduce migration. Additionally, Yin and colleagues have shown that CD44+ (high claudin-4) cells have an enhanced ability to migrate compared to the CD44- (low claudin-4) population of the same tumor [29]. These findings suggest, as with apoptosis, that claudin-4 likely interacts with key molecules at the plasma membrane to facilitate cell migration. We have previously shown, in normal epithelial cells, that DFYNP reduces tumor cell motility most efficiently when cells are grown on plates coated with type I collagen compared to fibronectin or the non-physiological adhesive Cell-Tak [27]. These observations suggest that claudin-4 may interact with proteins of the extracellular matrix, directly or indirectly, to promote motility. Recently, Fredriksson et al. published an elegant proteomics study examining the interacting partners of occludin and claudin-4 in MDCK II cells [30]. They found that claudin-4 interacts with CD44 and integrin beta-1, both cell surface proteins are known receptors for matrix proteins and are involved in promoting cell motility. An important question for future research is whether DFYNP acts by interfering directly with claudin-4 interactions with the extracellular matrix or by interfering with its interactions with other molecules that link tumor cells to the matrix.

Contrary to our observations, the Howell laboratory has shown that loss of claudin-4 expression in ovarian tumor cells enhances cell survival and inhibits migration [31]. This group has examined claudin-4 activity in the 2008 tumor cell line. Here, we have studied claudin-4 activity in SKOV3, OVCAR3, and PEO4 ovarian tumor cell lines. One significant difference between these cell lines is that 2008 cells do not express estrogen receptor alpha (ERα); whereas, the cell lines that we examined all express ERα. This suggests the possibility that claudin-4 may require ERα for activity. Alternatively, there is evidence that 2008 ovarian tumor cells may, in fact, be a contaminating cervical cancer cell line and this may explain the discrepancy in results [26].

## Conclusions

Results from this study provide key observations that support a functional role for claudin-4 expression in ovarian tumor cell survival and spread. Our hypothesis is that claudin-4 interacts with molecules at the tumor cell surface through extracellular loop interactions and alters intracellular signaling pathways to promote tumor cell survival and migration. Determining what claudin-4 interacts with at the cell surface and how claudin-4 alters cell signaling pathways are important next steps in understanding how claudin-4 regulates apoptosis and migration. Additionally, it will be important to understand whether claudin-4 promotes these same activities in normal epithelia, under tight regulation, and whether loss of this regulation or aberrant expression of claudin-4 may be an early event that leads to tumorigenesis.

## Abbreviations

cld4KD: Claudin-4 knockdown
contKD: Knockdown control (empty vector)
DFYNP: Aspartic acid-phenylalanine-tyrosine-asparagine-proline
DGYNP: Aspartic acid-glycine-tyrosine-asparagine-proline
